# Probing the Conformational Space of the Cannabinoid Receptor 2 and a Systematic Investigation of DNP-Enhanced MAS NMR Spectroscopy of Proteins in Detergent Micelles

**DOI:** 10.1021/acsomega.3c04681

**Published:** 2023-08-28

**Authors:** Johanna Becker-Baldus, Alexei Yeliseev, Thomas T. Joseph, Snorri Th. Sigurdsson, Lioudmila Zoubak, Kirk Hines, Malliga R. Iyer, Arjen van den Berg, Sam Stepnowski, Jon Zmuda, Klaus Gawrisch, Clemens Glaubitz

**Affiliations:** † Institute of Biophysical Chemistry and Centre of Biomolecular Magnetic Resonance, Goethe University Frankfurt, Max-von-Laue-Str. 9, 60438 Frankfurt, Germany; ‡ National Institute on Alcohol Abuse and Alcoholism, National Institutes of Health, Bethesda, Maryland 20852, United States; § Department of Anesthesiology and Critical Care, Perelman School of Medicine, University of Pennsylvania, Philadelphia, Pennsylvania 19104, United States; ∥ Department of Chemistry, Science Institute, University of Iceland, Dunhaga 3, 107 Reykjavik, Iceland; ⊥ Section on Medicinal Chemistry, National Institute on Alcohol Abuse and Alcoholism, National Institutes of Health, Bethesda, Maryland 20852, United States; # ThermoFisher Scientific, 7335 Executive Way, Frederick, Maryland 21704, United States

## Abstract

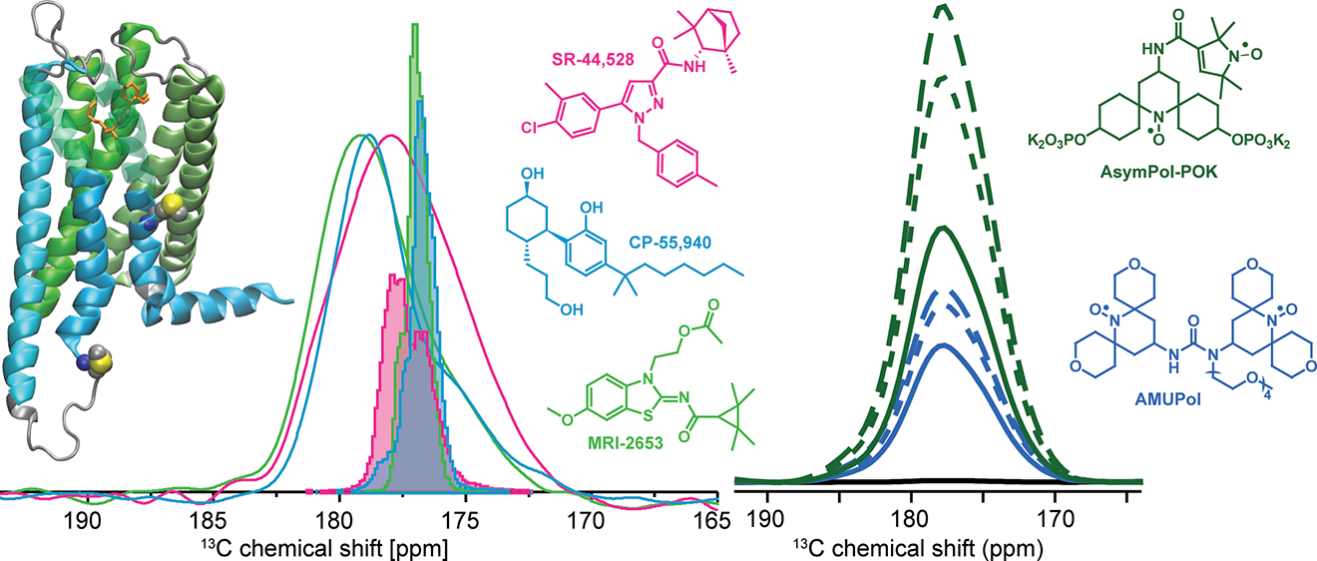

Tremendous progress has been made in determining the structures of G-protein coupled receptors (GPCR) and their complexes in recent years. However, understanding activation and signaling in GPCRs is still challenging due to the role of protein dynamics in these processes. Here, we show how dynamic nuclear polarization (DNP)-enhanced magic angle spinning nuclear magnetic resonance in combination with a unique pair labeling approach can be used to study the conformational ensemble at specific sites of the cannabinoid receptor 2. To improve the signal-to-noise, we carefully optimized the DNP sample conditions and utilized the recently introduced AsymPol-POK as a polarizing agent. We could show qualitatively that the conformational space available to the protein backbone is different in different parts of the receptor and that a site in TM7 is sensitive to the nature of the ligand, whereas a site in ICL3 always showed large conformational freedom.

## Introduction

The endocannabinoid system is involved in many physiological processes. The two human cannabinoid receptors, CB_1_ and CB_2_, belong to the class A G-protein coupled receptor (GPCR) protein family and share 44% amino acid similarity.^1^ CB_1_ is found in high concentrations in the brain but is also abundant in other tissues, whereas CB_2_ occurs in cells of the immune system and the nervous system. Therefore, both receptors are interesting pharmacological targets.^2^ Depending on the ligand, different signaling pathways are activated, and many ligands bind to both CB_1_ and CB_2_ receptors. To reduce off-target effects, the development of ligands that are highly selective for each receptor and the targeted signaling pathway is needed.^3^

Rational drug design requires structural information of the active and inactive states of the receptors as well as of the receptor/G-protein complexes. Tremendous progress in this area has been reported in recent years. CB_1_ X-ray structures have been determined in the antagonist^4^ and the agonist^5^ bound form as well as in the complex with the G-protein trimer.^6^ Following the work on CB_1_, CB_2_ structures in the antagonist bound state,^7^ the agonist bound state,^8^ and in the complex with the G-protein^8,9^ have been determined.

Despite the availability of this wealth of structural data on these and other GPCRs, the development of specific ligands has been difficult.^10^ One reason is the dynamic nature of GPCRs, which is not adequately captured by the available high-resolution structures.^11^ It has been shown by solution NMR that GPCRs are highly dynamic and even in the apo state, several active and inactive like conformations are sampled. Agonist and antagonist binding or G-protein coupling can shift these conformational equilibria or convert the system to conformations not populated in the apo state. It has been postulated that the differences in equilibrium between different active states are the origin of partial antagonism^11^ and it could also play a role in biased signaling.

In principle, solution-state NMR is a very valuable method to uncover such conformational equilibria. However, GPCRs in membrane memetic environments are challenging to study by solution NMR due to their size. Recently, ^19^F-NMR approaches have been developed to study GPCRs in solution.^12,13^ The unnatural amino acid 3′-trifluoromenthyl-phenylalanine has been used to study the effect of allosteric ligands on the distribution of both active and inactive conformations of CB_1_.^14^

However, ^19^F NMR is always associated with small but non-negligible changes to the primary sequence of the protein. The primary site of chemical introduction of ^19^F is cysteine residues, but they are not always accessible and often there are many cysteines in the sequence, resulting in difficulties in assignment. For example, the CB_1_ and CB_2_ receptors contain 13 cysteines each, which was one of the reasons to introduce an unnatural amino acid in the ^19^F NMR study on CB_1_.^14^ Similarly, in an EPR study of the dynamics of the intracellular loop 3 (ICL3) in CB_2_, it was difficult to obtain background free spectra as not all cysteine residues could be removed from the sequence and labeling efficiencies varied from site to site.^15^ In addition, although ^19^F NMR is very sensitive, dynamics on the intermediate NMR time scale can still result in extreme line broadening, rendering the signals non-detectable.

One way to suppress such dynamics would be to freeze the sample without restricting its conformational space by 3D crystallization or conformational selection, which happens due to image clustering during the analysis of cryo-EM data.^16^ In principle, solid-state MAS NMR on frozen samples can provide information on the whole conformational space.^17^ The linewidth and line shape of the NMR signal represent the conformational space that is available to the site of interest. The chemical shifts of the ^13^CO, ^13^Cα, and ^13^Cβ atoms strongly depend on the secondary structure of the respective amino acids and can be used to predict the backbone conformation.^18^ In a DNP study on the HIV capsid, NMR line shapes were recorded at low temperatures and compared with the dynamic ensemble from molecular dynamics MD trajectories.^19^ Due to the rigid nature of the HIV capsid, the NMR line shapes were relatively narrow. Low temperature spectra of GPCRs, which are very dynamic proteins, typically display broad lines and usually selective labeling schemes are applied.^20^

**Figure 1:**
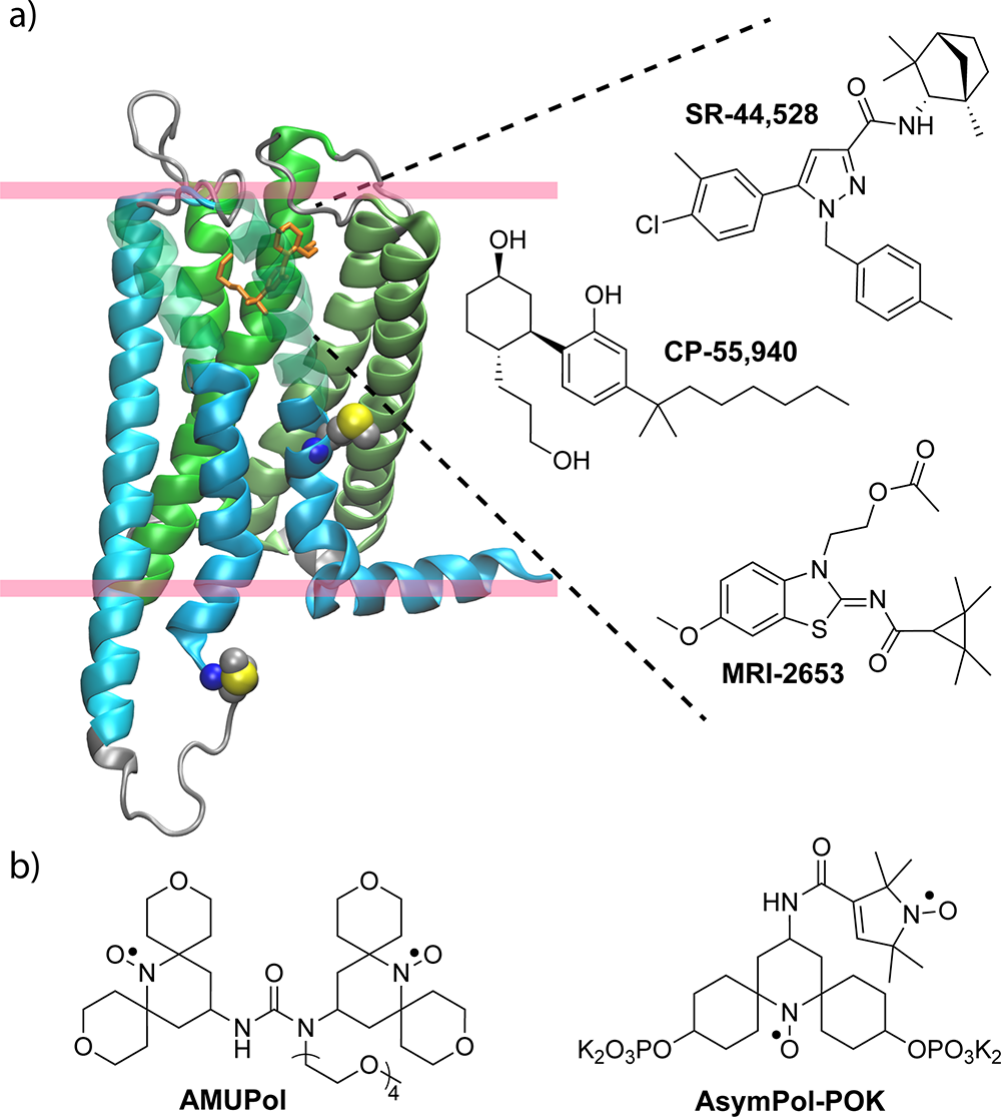
(a) Model of CB_2_ bound to the agonist CP-55,940 (orange) generated from PDB ID: 6PT0. The structure of CP-55,940 is shown together with the other two ligands used in this study: the antagonist SR-44,528 and MRI-2653, which act as partial agonists or partial inverse agonists, depending on the cholesterol content of the membrane. The unique pairs chosen for isotope labeling, M237R238 in ICL3 and M293V294 in TM7, are shown in a sphere representation. Pink bars indicate the position of the lipid headgroup region. (b) Polarizing agents: AMUpol and AsymPol-POK.

At low temperatures, NMR signal enhancement by dynamic nuclear polarization (DNP) can be used, reducing the amount of protein required. Therefore, application of MAS NMR to proteins that are difficult to obtain in large quantities, such as GPCRs, becomes possible. Unique pair labeling or other selective labeling strategies in combination with DNP signal amplification have been used, for example, to study the ABC transporter MsbA,^21^ the neuropeptide Y2 receptor, a class A GPCR,^20^ and Cytochrome-P450-Cytochrome-b5.^22^ In addition, some GPCRs have been studied by conventional solid-state MAS NMR methods, e.g., chemokine receptor 3,^23^ neuropeptide Y receptor 1,^24^ or rhodopsin.^25^ DNP enhancement requires the addition of a polarization agent to the sample. In recent years, the radical AMUPol (Figure 1b) has been the polarizing agent of choice when working with biological samples at moderate magnetic fields^26^ and has also been applied in the study of membrane proteins. In the past years, several new polarizing agents have been designed to further improve the DNP performance, e.g., by aiming at a better enhancement at higher magnetic fields or a reduction of the depolarization effect.^27^

Here, we show for CB_2_, in the presence of different ligands (Figure 1a), that insight into the conformational space of GPCRs can be obtained by DNP-enhanced MAS NMR. First, we devised an isotope labeling approach and then established the preparation of selectively labeled CB_2_ in highly concentrated micelles. We subsequently optimized the DNP protocols by varying the degree of deuteration of the DNP matrix and by using the new radical Asympol-POK^28^ (Figure 1b), which turned out to be advantageous for DNP experiments on frozen protein micelles. We then analyzed the shape and linewidth of the NMR signals at the different sites in the protein. In addition, we studied the protein in the presence of different ligands: the agonist CP-55,940, the antagonist SR-44,528 and MRI-2653, a ligand that can act as partial agonist as well as an antagonist, depending on the environment of the receptor.^29^ We could show that the conformational space differs between different sites in the protein and depends on the type of ligand. We employed molecular dynamics simulations (MD) to investigate if the motion observed during the MD runs is reflected in the experimental line shapes. We expect our approach generally to be useful for analyzing the conformational space of proteins even in cases where dynamics hinder experiments at ambient temperatures.

## Methods and Materials

### KR2 Protein Expression and Purification

U-^13^C,^15^N-KR2 samples were prepared as described previously.^30^ Briefly, wild-type KR2 was transformed in *E. coli* C43 (DE3). After preculture in LB medium, the cells were transferred into M9 medium supplemented with ^13^C_6_-glucose and ^15^NH_4_Cl. The culture was grown at 37 °C until OD_600_ = 0.6. Expression was induced with 0.5 mM IPTG and 7 μM all-*trans* retinal and was carried out over night at 27 °C. After harvesting and cell disruption, the membranes were solubilized with 1.5% (w/w) dodecyl-β-D-maltoside (DDM) and the protein was purified by Ni-NTA affinity chromatography.

### CB_2_ Expression Construct

Full length non-codon optimized CB_2_ ((NP_001832.1) amino acids 1–360/bp 163–1245 of NM_001841.3), N-terminally twin-Streptag and C-terminally 10-HIS tagged was synthesized and cloned in the pcDNA5-TO backbone (ThermoFisher) by GeneART. The nucleotide sequence of the construct is provided in the Supplemental Information (Figure S1).

### Expression of CB_2_ in Expi293GNTI- Cells

Maintenance of Expi293FGNTI- cells and optimization of CB_2_ expression in Expi293GNTI- cells were previously described.^31^ For the expression of methionine/valine and methionine/arginine-labeled CB_2_, custom amino acid-depleted Expi293 expression media and complexation medium lacking the aforementioned amino acids were manufactured by ThermoFisher. Briefly, Expi293F GNTI- cells (ThermoFisher cat. A39240) were grown in complete Expi293 expression medium at 37 °C with ≥80% relative humidity and 8% CO_2_ on a 19 mm orbital shaker at 125 rpm to a density of about 4 × 10^6^/mL and reseeded at 2.5 × 10^6^/mL one day prior to transfection. At the day of transfection, cells were counted, centrifuged (1000 g × 5 min), and resuspended in amino acid-depleted medium in 400 mL at a density of 3 × 10^6^/mL in 2 L shake flask. Six hours after amino acid starvation, ^13^C_5_-Met and ^15^N_4_-Arg (or ^13^C_5_-Met and ^15^N-Val) were added from a 100× stocks to the manufacturer’s specified concentration. At the same time, ligands were added from 10 mM stock solutions in DMSO: MRI-2699 – to final concentration 2.5 μM (or SR-144,528 – to final concentration 5 μM; or CP-55,940 – to final concentration 5 μM) into the cell culture. Immediately following medium supplementation, cells were transfected as per the manual with a slight modification. Briefly, for 400 mL of cell suspension, 1.3 mL of Expifectamine293 was diluted in 46.5 mL of amino acid- depleted Optiplex, incubated for 5 min before 400 μL of 1 mg/mL CB_2_-expression plasmid was added. This mixture was further incubated for 5 min before addition to the 2 L culture flask. After transfection, cells were incubated for 18 h before 2.4 mL of enhancer 1 was added. No enhancer 2 was added. Cells were harvested 48 h post-transfection by centrifugation, and cell pellets were stored at −80 °C until purification.

### Purification of CB_2_

Upon partial thawing, cells were resuspended in Tris-buffered saline (TBS) solution with Complete Protease Inhibitor (no EDTA), DNAse I, and 5 mM MgCl_2_. Cells were lysed using a cell homogenizer (Avestin). Upon homogenization, stock solution of an appropriate ligand (CP-55,940, SR-144,528, or ^13^C-MRI-2653 – with a ^13^C label on the methoxy group) in DMSO to the final concentration of the ligand of 5 μM, and Buffer A (2) × solubilization buffer: 100 mM Tris- HCl (pH 7.5), 300 mM NaCl, 60% glycerol; and 10 × “Triple detergent” solution (1.2% CHS, 10% DDM, 6% CHAPS) were added and the resulting solution was stirred for 1 h at 4 °C. After stirring, the crude cell extract was centrifuged at 215,000*g* for 1 h to separate out the solubilized protein. The supernatant was applied to Dowex 1 × 4–50 ion exchange resin for 30 min with orbital mixing at 4 °C. The resin was removed from the protein solution on a 0.45 mm filter. The resulting extract was further purified using tandem Ni- NTA and StrepTactin XT affinity chromatography. Using an AKTA-Purifier FPLC system, the protein solution was applied and washed on a 5 mL prepacked Ni-NTA column directly followed by a 5 mL prepacked StrepTactin XT High-Capacity column to capture any non-bound protein from the Ni-NTA column. The protein was eluted from the Ni-NTA resin using imidazole elution buffer (10 μM ligand; Buffer A; 250 mM imidazole) and collected on the StrepTactin XT resin.

### Detergent Exchange and Sample Preparation

Once all proteins were bound to the StrepTactin XT column, detergent exchange was performed. The protein solution was washed with Buffer A supplemented with 10 μM ligand. The detergent was then exchanged from Buffer A to FA buffer (0.5–1.0% DMSO, 0.25 mM Façade-TEG/CHS buffer, 20 mM HEPES; pH 7.5, 10 μM corresponding ligand) using gradient washes. After detergent exchange, the protein was eluted from the StrepTactin XT column using FA elution buffer (FA buffer with 20 mM biotin). Protein fractions were collected and washed several times using deuterated Tris buffer (Sigma Aldrich Cat 486248) supplemented with FA/CHS and 10 μM corresponding ligand dissolved in *d*_6_-DMSO and then concentrated using Amicon centrifugal concentrators. The resulting protein concentration was measured using the DCA protein assay (BioRad Laboratories). Finally, to 15 μL of the concentrated protein solution, 15 μL of ^13^C-depleted *d*_8_-glycerol (Cambridge Isotope Laboratories, cat. CDLM-8660-PK) was added, and the protein samples were frozen in liquid nitrogen. The protein concentration in these samples was 0.25–0.5 mM.

### DNP Sample Preparation

AMUPol was obtained from Cortecnet, and AsymPol-POK was prepared as described previously.^28^

1-^13^C-glycine samples for DNP experiments were prepared using a stock solution of 2.36 M 1-^13^C-glycine and 50 mM AMUPol or AsymPol-POK, respectively. 2.5 μL of this solution was then mixed with the appropriate amount of H_2_O, D_2_O, and ^2^H_8_-glycerol or glycerol, see Table S1. The final concentration of the polarizing agent was 5 mM. The samples were packed into 3.2 mm sapphire MAS NMR rotors and confined to the center with a Teflon top insert before closing the rotor with a Vespel cap. Each sample contained 5.9 μmol 1-^13^C-glycine. MW on/off enhancements were measured by comparing spectra, which were recorded at 5 times *T*_1_(^1^H); the measured *T*_1_(^1^H) times are listed in Table S1.

For the systematic study on KR2, to reduce pipetting errors and to obtain identical amounts of protein for each sample, the purified KR2 micelles were concentrated with a cut-off of 100 kDa in one batch to a concentration of 20 mg/mL and then mixed with ^2^H_8_-glycerol at a ratio of 1:1 (v/v). 30 μL of the mixture was then used directly or added to AsymPol-POK or AMUPol to yield 7 samples with the following polarizing agent concentrations: 0 mM AMUPol/AsymPol-POK, 5 mM AMUPol, 10 mM AMUPol, 20 mM AMUPol, 5 mM AsymPol-POK, 10 mM AsymPol-POK, 20 mM AsymPol-POK. As the amount of radical used for each sample was very small and would have been difficult to weigh, we used the following procedure. Stock solutions of AMUPol (20 mM in CHCl_3_) and Asympol-POK (6.562 mM in CHCl_3_:methanol 2:1 (v/v)) in organic solvent were prepared. The required amount of dissolved polarizing agent was then added to a sample tube, and the solvent was removed under a gentle steam of nitrogen. The protein mixture was then added to the dried radical, which then dissolved. The samples were packed in 3.2 mm sapphire MAS NMR rotors and confined to the center with a small Teflon top insert before closing the rotor with a Vespel cap. Unfortunately, the rotor with the 10 mM AsymPol-POK sample cracked when adding the rotor cap and the sample had to be repacked to another rotor. This procedure led to a slight loss of sample, which we estimate to be 10%.

CB_2_ DNP-samples at a final concentration of 0.25–0.5 mM were prepared in a similar way as described for KR2 and contained 10 mM AsymPol-POK. For these samples, to suppress natural abundance signals from glycerol, deuterated ^13^C-depleted glycerol (^2^H_8_,^12^C_3_-glycerol) was used.

### DNP Experiments

All experiments were recorded on a 400 MHz WB NMR spectrometer equipped with a 3.2 mm DNP Cryo probe. Microwaves were generated with a 263 GHz Bruker gyrotron. All experiments were carried out at the lowest temperature available (100–105 K) at a MAS spinning frequency of 8 kHz. The experiments were carried out with 100 kHz ^1^H SPINAL decoupling, and if not mentioned otherwise, the recycle delay was 1.3 × ^1^H-*T*_1_ to maximize the signal-to-noise per time. ^13^C and ^15^N CP experiments were done using an 80–100% ramp on the ^1^H channel and contact time of 800 μs. The selective CP transfer in the NCO, NCOCX, and CON experiments was done using a 90–100% ramp on the ^13^C channel with a contact time of 4 ms. The COCX step in the NCOCX experiments was done using a 20 or 50 ms proton driven spin diffusion mixing time (50 ms was used for MR^ICL3^-CB_2_ + CP and MR^ICL3^-CB_2_ + MRI but then lowered to improve Cα sensitivity, which did not affect the line shape). The double quantum filtered experiment was recorded using Post-C7. ^1^H-T_1_ was measured using a saturation recovery experiment. ^13^C-*T*_2_ and ^15^N-*T*_2_ were measured with the Hahn echo pulse sequence. The number of acquisitions (ns) of the individual spectra are given in the corresponding figure captions. The sensitivity for the KR2 samples was measured by recording the spectra during 10 min with a recycle delay of 1.3 × ^1^H-T_1_, resulting in 30, 62, 100, 190, 236, 490, and 728 scans for the samples with no polarizing agent, 5 mM AMUPol, 10 mM AMUPol, 20 mM AMUPol, 5 mM AsymPol-POK, 10 mM AsymPol-POK, and 20 mM AsymPol-POK, respectively. KR2 samples were referenced to the left natural abundance signal of glycerol at 64.78 ppm with respect to DSS. The CB_2_ samples contained the Façade detergent with a typical sugar signal. This signal at 105.2 ppm with respect to DSS was used for referencing.

### Data Analysis

**Figure 2:**
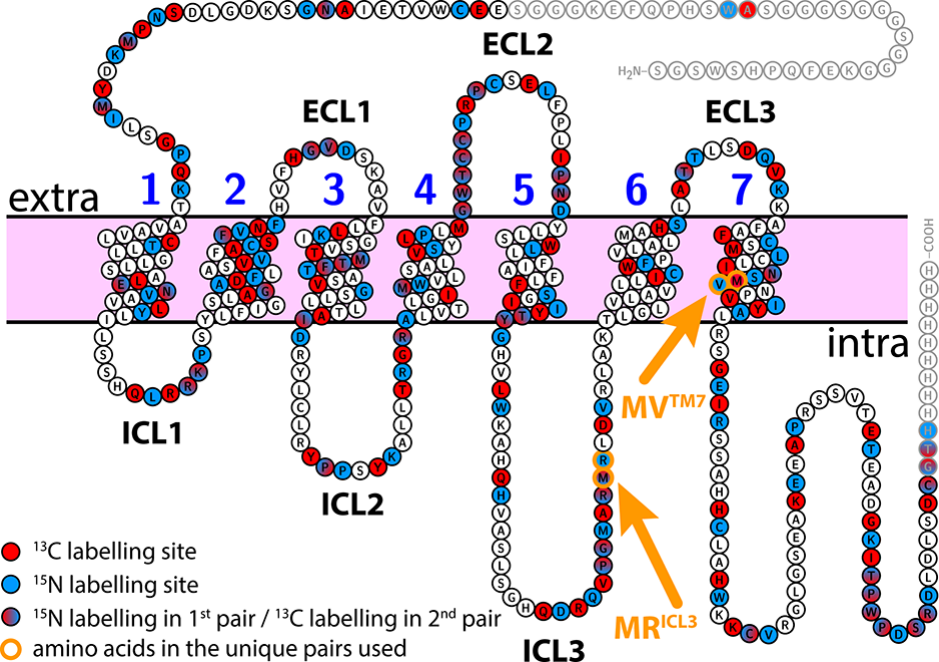
Visualization of the unique pairs M237-R238 (MR^ICL3^) and M293-V294 (MV^TM7^) in CB_2_. Amino acids that are part of a unique pair are colored in blue and red. The unique pairs used in the present work are highlighted in orange.

Visualization of the unique pair distribution in CB_2_ (Figure 2) was done based on the output from Protter et al.^32^ For fitting of *T*_1_ and *T*_2_ times, spectra were integrated using Topspin 4.1.3 and then imported into OriginPro 2017 for further analysis. *T*_1_ and *T*_2_ times were obtained from monoexponential fits, and in the case of the ^13^C-*T*_2_ (CO), the ^1^J(CO,Cα) coupling was also taken into account and was fixed at 50.7 Hz. Methionine chemical shifts were analyzed using PLUQ.^18^

### MD Simulations

We followed the same simulation procedures as in previous work^29^ for generating simulation parameters for the ligands. Each ligand was geometry-optimized using the B3LYP/6-31G** quantum mechanics level of theory and basis set using Gaussian09.^33^ The CHARMM36 force field^34^ was used for all molecular dynamics simulations. Ligand parameters starting from the geometry-optimized ligands structures were derived from CGenFF;^34^ these high-affinity ligands were not expected to deviate significantly from their original bound configurations.

The CB_2_ structure starting point was a cryo-EM structure (Protein Data Bank: 6PT0).^9^ This was oriented in a lipid membrane using the Orientations of Proteins in Membranes (OPM) database^35^ using the CHARMM-GUI input generator.^36^ The lipid membrane contained 40% cholesterol and POPC:POPG in a 3:1 ratio. Sodium and chloride ions were added to 0.15 M plus excess ions for electroneutrality. The cholesterol molecules present in the cryo-EM structure were retained. The system was minimized and equilibrated with side chain and backbone restraints, which were then released. Production simulations were run in the isothermic-isobaric ensemble at 303.15 K using NAMD 2.13^37^ with GPU extensions. Particle Mesh Ewald summation of long-range interactions was used, as were the Langevin barostat and thermostat.

## Results and Discussion

### Unique Pair Labeling Approach

Solid-state NMR experiments on proteins usually require the enrichment of NMR active nuclei such as ^13^C and ^15^N in the sample. To circumvent resolution problems that arise from the frozen state in combination with the size of CB_2_ and to facilitate the assignment, we applied the unique pair labeling technique:^38^ A unique pair in a protein are two neighboring amino acids that are only found once in the primary sequence. One of the amino acids is ^15^N labeled, and the other one is ^13^C labeled to give a single correlated NMR signal. Given the total number of 400 different amino acids pairs, a protein of the size of a GPCR usually contains a significant number of unique pairs and it is likely to find such a pair in the vicinity of the site of interest, e.g., switches, ortho or allosteric binding pockets, G-protein or arrestin binding interfaces.

Analysis of the sequence of our CB_2_ construct revealed 113 unique pairs, distributed over the whole sequence (Figure 2). Active and inactive GPCR conformations differ in the orientation of the intracellular half of helix 6 and in ICL3. During activation, the hydrophobic lock between transmembrane helix 3 (TM3) and TM6 loosens and enables the outward movement of TM6.^39^ To probe the dynamic in this region, two unique pairs are available, M237-R238 and D240-V241. We picked the first one for selective isotope labeling as asparagine often suffers from isotope scrambling, depending on the expression system. A similar position has been used for ^19^F labeling in a study on β_2_AR and was shown to be sensitive to activation of the protein.^40^ Therefore, a ^13^C_5_-methionine/^15^N_4_-arginine labeled CB_2_ sample was prepared to create a ^13^C-M237-^15^N-R238 unique pair. In the following, we refer to this sample as MR^ICL3^-CB_2_. In addition, we selected ^13^C_5_-methionine and ^15^N-valine to obtain the ^13^C-M293-^15^N-V294 unique pair, which is located in the middle of TM7, and we refer to it as MV^TM7^-CB_2_. This position is far away from the orthosteric binding site and thus, should not be directly sensitive to binding of different ligands (Figure 1a). The residue pair is next to the NPxxY motif conserved in class A GPCRs^39^ and should reflect changes in the transmembrane region upon ligand binding.

### Expression and Sample Preparation of Selectively Labeled CB_2_

Preparation of the MR^ICL3^-sample and MV^TM7^-sample requires a protocol that is compatible with selective isotope labeling. Previously, metabolic labeling of the CB_2_ receptor with stable isotopes has been achieved by fermentation of *E. coli* in minimal medium, supplemented with labeled nutrients.^41^ However, the bacterial expression of the receptor did not allow for important post-translation modifications (glycosylations) that, as some of us have recently demonstrated, are important for stability of CB_2_ isolated in detergent micelles.^42^ Here, CB_2_ was selectively labeled by expression of the receptor in a suspension of Expi293GNTI- cells that allow for controlled glycosylation of CB_2_, following our previously published protocol, which yielded protein showing G-protein activation.^31^ The expression of ^13^C_5_-methionine/^15^N-valine and ^13^C_5_-methionine/^15^N_4_-arginine labeled CB_2_ was performed in custom amino acid-depleted Expi293 expression media and complexation medium lacking the aforementioned amino acids. The yield of labeled receptor was on the order of 2–3 mg/L of culture, which is on par or better than previously reported levels of expression of isotope-labeled GPCR in other expression systems (*E. coli*, yeast *Pichia pastoris*).^12,43^

Preparation of membrane proteins for DNP-enhanced MAS NMR measurements requires high concentrations of active protein in a membrane mimetic, such as detergents, nanodiscs, or liposomes. Procedures that embed the protein in nanodiscs or liposomes are often prone to loss of protein during the reconstitution procedure.^44^ To avoid loss of valuable isotope labeled protein, we decided to prepare CB_2_ in a detergent. We have previously demonstrated that Façade detergents are equal or even superior to other detergents in their ability to stabilize the functional structure of CB_2_.^15,42^ We supplemented the Façade-TEG detergent with cholesterol derivative CHS to stabilize the receptor.^42^ The low aggregation number of Façade-TEG allows concentration of the protein sample to 0.5 mM, or even higher, without accompanying co-concentration of the detergent, which was mandatory due to the limited sample volume available in the MAS NMR rotors (here, 30 μL). We could show that during this concentration step, the receptor kept the ability to bind ligands by detecting the ^13^C-signal of ^13^C-MRI-2653 in our preparations, see Figure S2.

In order to find optimal conditions for DNP-enhanced ssNMR experiments, which are compatible with the CB_2_ sample requirements, we describe in the following a systematic study on the role of the DNP matrix composition and choice of polarizing agents.

### Effects of the Matrix on DNP Enhancement and Sensitivity

Enhancement techniques are mandatory to obtain single-atom sensitivity on sub-mg quantities of GPCRs in the frozen state. Due to the low temperatures employed, DNP of the NMR active nuclei is the method of choice. This requires the presence of a polarizing agent, typically a biradical in a frozen glassy matrix. For aqueous solutions, a mixture termed “DNP juice” (10% H_2_O, 30% D_2_O, 60% ^2^H_8_-glycerol vol%) was suggested many years ago^45^ and has since then been used frequently.^27^ Also the usage of a matrix using ^2^H_6_-DMSO instead of ^2^H_8_-glycerol has been described^46^ as well as matrix optimizations in impregnated materials and in-cell experiments.^47,48^ For proteoliposome samples, an incubation approach has been established and optimized.^49^ Systematic studies on the degree of deuteration and the glycerol content in aqueous solutions are only available in early studies not using modern biradicals.^50^ In a study on the human bradykinin 2 receptor, a glassy matrix made from 10% H_2_O, 40% D_2_O, and 50% ^2^H_8_-glycerol was used,^51^ slightly deviating from the “DNP juice”. The large amount of glycerol in the “DNP juice” reduces the space available in the rotor for the sample of interest, which is usually dissolved in water. In addition, the high degree of deuteration of the aqueous phase requires an additional buffer changing step during sample preparation, risking loss of valuable protein, which should be avoided when working with samples that are only available in limited amount or have a limited stability or both. To be able to estimate the effects of the glycerol content and the degree of deuteration on DNP-enhancements, we carried out a systematic study of these two parameters.

**Figure 3:**
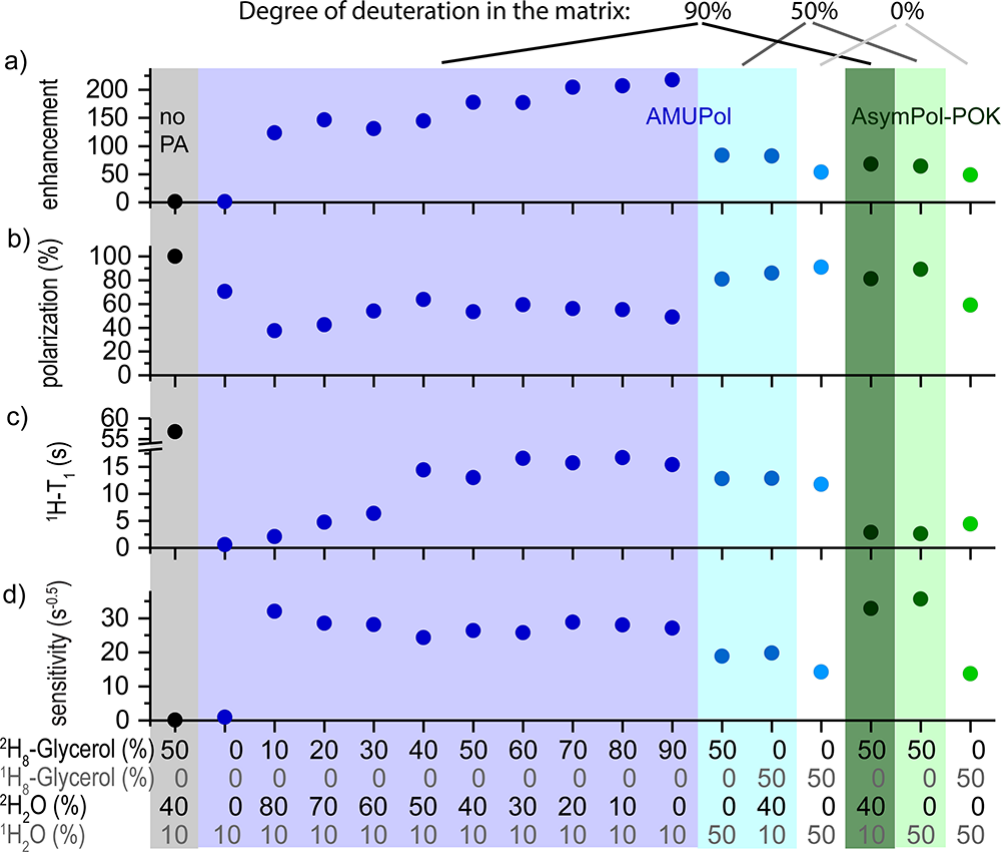
DNP and relaxation parameters of 0.24 mM 1-^13^C-glycine in different DNP matrices with varying H_2_O/D_2_O/glycerol ratios. Shown in black is the data on a sample without any radical. Samples in dark blue contained 5 mM AMUPol in different deuterated matrices. Samples in blue have a reduced degree of deuteration, and the light blue samples is in a fully protonated matrix. The samples shown in green contain 5 mM AsymPol-POK, dark green: deuterated matrix, green: reduced degree of deuteration, light green: fully protonated. (a) Microwave on/off enhancement. (b) Polarization compared to the sample without radical. (c) *T*_1_(^1^H) relaxation times in the presence of microwave. (d) Sensitivity.

We chose 1-^13^C-glycine in the presence of 5 mM AMUPol for this study because glycine can be dissolved at high concentrations. To make sure that the AMUPol and 1-^13^C-glycine concentrations within the experimental series are exactly the same, a stock solution of 2.36 M 1-^13^C-glycine and 50 mM AMUPol in water was prepared. Then, the appropriate amounts of H_2_O, D_2_O, and ^2^H_8_-glycerol or ^1^H-glycerol were added to obtain the sample of choice. Due to the limited solubility of AMUPol in the stock solution, this procedure resulted in a total concentration of 5 mM AMUPol in the samples, slightly lower than the value of 10 mM typically found in the literature. We expect the observed trends to be very similar when using higher AMUPol concentrations. In addition, a sample without polarizing agent (no PA) was prepared in a 1:4:5 H_2_O:D_2_O:^2^H_8_-glycerol mixture. The DNP and relaxation parameters obtain on these and samples with AsymPol-POK, described below, are given in Table S1 and shown in Figure 3.

Figure 3a–c shows the microwave (MW) on/off-enhancements, the polarization, and the *T*_1_(^1^H) relaxation times, obtained on the experimental series, respectively. The polarization was determined by comparing the signal intensity of the microwave-off spectra, recorded with a recycle delay of five times *T*_1_(^1^H), with the signal intensity of the sample without the polarizing agent. This way, we account for MAS-induced interference as well as paramagnetic bleaching due to the biradical. First, we compared samples with a high degree of deuteration (90%) but different water/glycerol ratios (dark blue data points in Figure 3). In the sample without glycerol (0%), no enhancement was obtained. Due to the relatively slow freezing after the insert of the sample into the pre-cooled probehead, no glassy matrix was obtained in a pure water sample, thus preventing efficient signal enhancements. Interestingly, although no enhancement was obtained, reduced polarization was observed. *T*_1_(^1^H) was very short in this sample, the shortest of our series, also indicating the absence of a glassy matrix. When D_2_O was then stepwise replaced by ^2^H_8_-glycerol, significant enhancements were obtained. Even the presence of only 10% ^2^H_8_-glycerol resulted in a 123-fold enhancement. This value increased gradually with the ^2^H_8_-glycerol concentration until an enhancement of 217 was reached in the sample with 90% ^2^H_8_-glycerol. Depolarization is induced in the sample by the polarizing agent and the lowest polarization was observed at 20% ^2^H_8_-glycerol. Above this concentration, the polarization was between 50 and 60%, showing no strong matrix dependency. *T*_1_(^1^H)-times, which were shortest in the absence of glycerol, increased with increasing amounts of ^2^H_8_-glycerol (Figure 3c). Here, *T*_1_(^1^H)-times measured in the presence of the microwave are shown, but very similar values were obtained, when *T*_1_(^1^H)-times were measured without microwave irradiation, see Table S1. Above 30% ^2^H_8_-glycerol, an increase in *T*_1_(^1^H)-times was observed, which then ranged between 13 and 17 s. We attribute this sudden change in *T*_1_(^1^H) when going from 30% ^2^H_8_-glycerol to 40% to a change in proton dynamics in the sample due to the formation of a glassy matrix upon freezing the sample. Interestingly, this does not coincide with the sharp increase observed in enhancement, which occurs already at 10% ^2^H_8_-glycerol. Such a low amount of glycerol might be sufficient to ensure a good distribution of the polarizing agent in the matrix, possibly by preventing aggregation upon freezing. However, at this very low ^2^H_8_-glycerol concentrations, a large loss of polarization is observed and a higher amount of glycerol is needed to prevent strong depolarization.

Enhancement, polarization, and also the *T*_1_(^1^H)-time, which dictates the recycle delay of an experiment, all contribute to the signal-to-noise per time that can be recorded on a specific sample. To account for these factors, we calculated the sensitivity as used by Mentink-Vigier et al. (2018) and in earlier studies:^28,48,52,53^
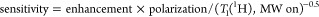


The results are shown in Figure 3d and given in Table S1. Interestingly, the sample with the highest sensitivity was the sample with only 10% glycerol due to the very fast *T*_1_(^1^H) and the moderate depolarization observed in this sample, which compensates for the relatively low enhancement. However, the sensitivity calculated for the other samples of the series was not much lower. In samples with protein instead of glycine, we expect intrinsically lower *T*_1_(^1^H) times due to methyl group rotations that are still active in the temperature range around 100 K. Thus, the advantage of the faster relaxation at low glycerol concentrations might be less important for samples with protein. In addition, glycerol acts as a cryoprotectant when it forms a glassy matrix, which happens in our samples at concentrations of 40% and higher, as seen from the *T*_1_(^1^H) times. For proteins with limited stability, it is therefore recommended to have at least 40% glycerol in the sample. At higher glycerol concentrations, the enhancements and sensitivity increase slightly, but the more glycerol is used during sample preparation, the smaller the volume available for the sample of interest. Considering all these factors, we decided to prepare all further samples in this study in 50% glycerol as this is well above the threshold for the formation of a glassy matrix and still 50% of rotor volume can be used for the sample of interest, a 25% larger fraction compared to the traditional DNP mixture.

Next, we investigated the degree of deuteration of the glassy matrix. Three samples were prepared with a reduced degree of deuteration by either replacing D_2_O with H_2_O, ^2^H_8_-glycerol with glycerol, or both (blue and light blue data points, respectively, in Figure 3). The enhancement drops significantly in these samples, but less magnetization is lost due to depolarization. *T*_1_(^1^H) times are not affected significantly. Due to the low enhancements, the sensitivity in these samples is clearly reduced and the effect is most pronounced for the fully protonated sample.

In addition to the MW on/off-enhancements, a large contribution to the sensitivity is the polarization, which is strongly affected by AMUpol, and *T*_1_(^1^H) times. We therefore replaced AMUPol by AsymPol-POK, green in Figure 3. This radical has been described by Mentink-Vigier et al.^28^ and shown to give better sensitivity than AMUPol due to reduced depolarization and higher relaxivity, which compensated for the observed lower enhancements. Here, we also obtained a lower enhancement compared to AMUPol, but more importantly we observed an increased sensitivity due to reduced depolarization and higher relaxivity.

AsymPol-POK has also been shown to be much more tolerant toward high degrees of protonation.^54^ We see the same trend in our samples and going from the highly deuterated matrix to a matrix where only the glycerol is deuterated did not change the DNP performance significantly (green in Figure 3). However, using a fully protonated matrix resulted in a drop in sensitivity (light green in Figure 3). This situation could perhaps be further improved by using cAsymPol-POK, an AsymPol-POK derivative, which is even more tolerant against high proton densities.^54^ In practice, in our and most other samples, this is not crucial as sample preparation is not compromised by using ^2^H_8_-glycerol in instead of protonated glycerol.

### DNP Enhancement and Sensitivity in Frozen Detergent Micelles

Optimizing the sample conditions also requires fine-tuning of the radical concentration as shown in an early study by Tycko et al.^52^ In addition, it is also of interest to quantify if coherence lifetimes are affected by the presence of the polarizing agent, a point which is often overlooked. Ideally, optimization of the radical concentration is performed directly on the sample of interest. However, systematic studies require a large amount of material, which is difficult to obtain on challenging systems such as GPCRs. We therefore used KR2, a hepta-helical transmembrane protein from the microbial rhodopsin family with similar size and topology as CB2. It is expressed well in *E. coli* and can easily be isotope-labeled,^55^ to investigate the difference between AMUPol and AsymPol-POK and to find their best concentration. KR2 was uniformly ^13^C,^15^N-labeled and prepared in DDM detergent micelles. We did not perform a buffer exchange to a deuterated buffer as we wanted to avoid such a buffer exchange in the experiments on the GPCR, to avoid loss of sample or activity.

**Figure 4:**
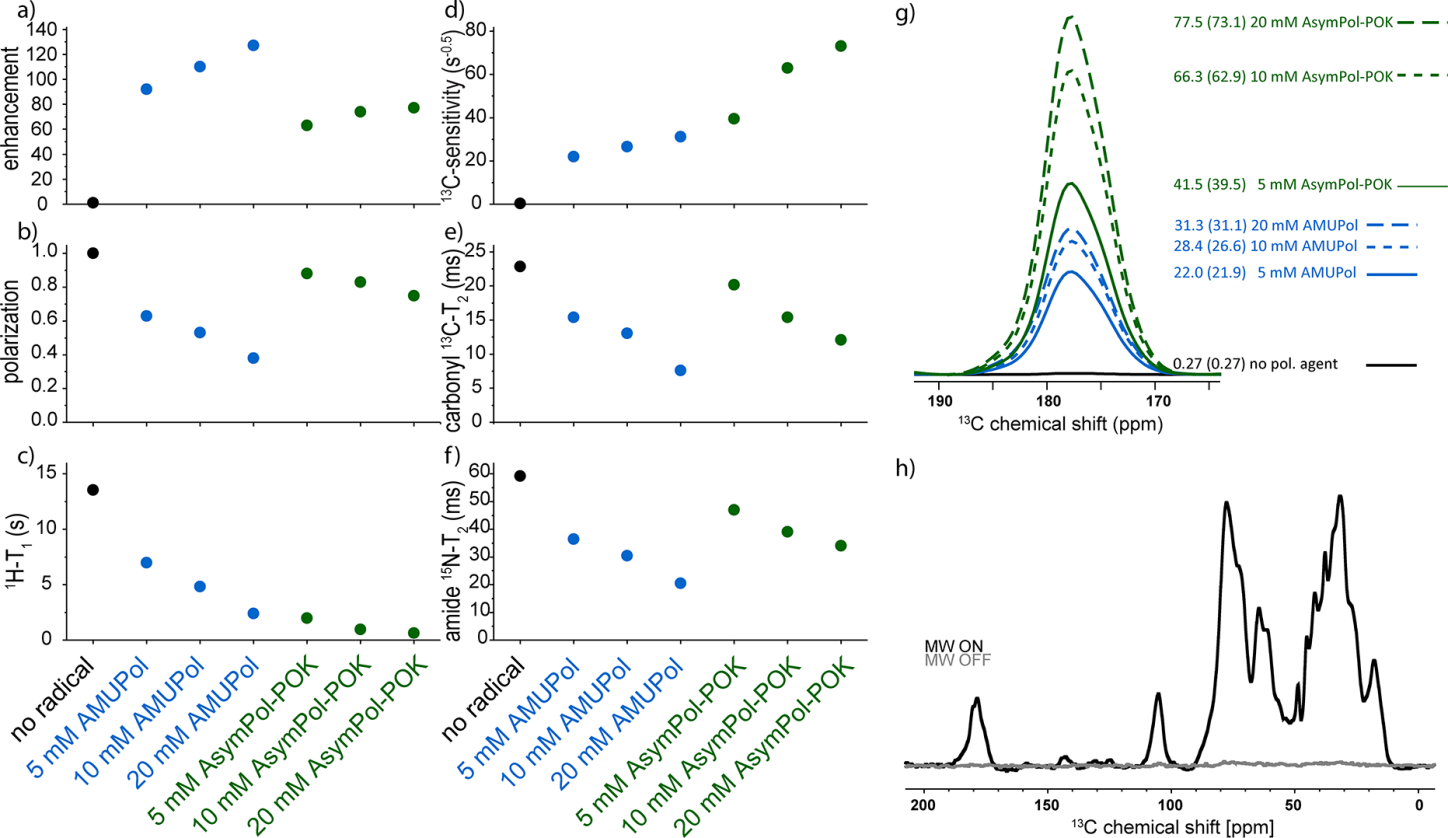
Concentration-dependent DNP and NMR parameters of KR2-DDM. (a) Enhancement, (b) polarization, (c) *T*_1_(^1^H) (MW on), (d) carbonyl-^13^C sensitivity, (e) carbonyl-^13^C-T_2_, (f) amide-^15^N-T_2_, and (g) carbonyl region of the ^13^C cross-polarization spectra on KR2-DDM with different polarizing agents. Each spectrum was recorded during an experimental time of 10 min with a recycle delay corresponding to 1.3 ^1^H-*T*_1_. The spectra were scaled to show the same noise. The numbers in the plot shown are measured relative signal intensity whereby the intensity of the sample without polarizing agent was set to 0.27 to correspond to the calculated sensitivity in Table 1. For comparison, the number in brackets give the calculated sensitivity from Table 1 in s^–0.5^, (h) microwave (on/off) spectra of MV^TM7^-CB_2_ + CP-55,940, microwave (on/off) enhancement = 66.

Seven different samples were prepared, three with varying concentrations of AMUPol and AsymPol-POK, respectively, and one without any polarizing agent. Special care was taken to add the exact same amount of protein to each sample to be able to quantify the depolarization. This was accomplished by preparing one batch of a 50:50 (v/v) mixture of ^2^H_8_-glycerol and KR2-DDM solution that for each sample was added in the same amount to the different radicals and packed into 3.2 mm sapphire MAS rotors. We subsequently measured ^1^H-*T*_1_ (with and without microwave irradiation), carbonyl-^13^C-*T*_2_, amide-^15^N-*T*_2_ (using the Hahn Echo experiment), and recorded spectra with and without microwave with 5 times *T*_1_(^1^H) for quantitative analysis. The enhancement was determined by comparing the intensity of the signals with and without microwave irradiation at a recycle delay corresponding to 5 times *T*_1_(^1^H). The depolarization was obtained by comparison of the observed intensity of the sample containing the polarizing agent to the sample without the polarizing agent at 5 times *T*_1_(^1^H) without microwave irradiation. The sensitivity was calculated using this data and is shown together with the other parameters in Table 1 and Figure 4.

**Table 1:** Relaxation, Enhancement, Polarization, and Sensitivity Measured on KR2-DDM at Different Concentrations of the Polarizing Agents

sample	*T*_1_(^1^H) (MW on) (s)^b^	*T*_1_(^1^H) (MW off) (s)^b^	amide-^15^N-*T*_2_ (ms)	carbonyl-^13^C-*T*_2_ (ms)^c^	enhancement (MW on/off)	polarization	sensitivity
no polarizing agent	–	13.5 ± 1.2	59 ± 6	23 ± 2	1	100%	0.27 s^–0.5^
5 mM AMUPol	7.0 ± 0.2	6.5 ± 1.0	36.4 ± 1.0	15.4 ± 0.6	92	63%	21.9 s^–0.5^
10 mM AMUPol	4.8 ± 0.1	4.0 ± 0.4	30.5 ± 1.2	13.0 ± 0.4	110	53%	26.6 s^–0.5^
20 mM AMUPol	2.4 ± 0.1	2.0 ± 0.2	20.5 ± 1.2	7.6 ± 0.8	127	38%	31.1 s^–0.5^
5 mM AsymPol-POK	2.0 ± 0.1	2.3 ± 0.1	47.0 ± 0.8	20.2 ± 2.0	63	88%	39.5 s^–0.5^
10 mM AsymPol-POK	1.0 ± 0.1	1.0 ± 0.1	39.1 ± 0.4	15.4 ± 1.0	74	83%^a^	62.9 s^–0.5^
20 mM AsymPol-POK	0.6 ± 0.1	0.7 ± 0.1	34.1 ± 0.4	12.1 ± 0.7	77	75%	73.0 s^–0.5^

a Rotor had to be repacked due to a crack after closing of the rotor cap. We estimate 10% sample loss. This has been accounted for in the value of 83%.

b *T*_1_(^1^H) times were determined on the ^13^C–CO signal.

c Fitting of the carbonyl Hahn echo decay was done by taken a ^1^J_COCα_ coupling of 50.7 Hz into account. Errors correspond to the ≥95% confidence interval.

The observed enhancements are strongly concentration-dependent and the highest value of 127 was observed in the sample with 20 mM AMUPol (Figure 4a). Similarly, the polarization depended strongly on the radical concentration. A loss of signal intensity due to depolarization, based on the MAS-dependent transfer of magnetization from the nuclei to the electrons in the absence of microwave, and due to paramagnetic signal bleaching was observed for both radicals (Figure 4b). Comparing the radicals, AsymPol-POK led to significantly reduced depolarization and even the samples with the highest tested AsymPol-POK concentration (20 mM) had a higher polarization under MAS than the sample with just 5 mM AMUPol.

*T*_1_(^1^H) times in the absence of a polarizing agent are much shorter than in the glycine sample described above due to the dynamics of the protein that are still present around 100 K, e.g., methyl group rotations. In the presence of the biradical, a concentration-dependent reduction of *T*_1_(^1^H) was observed due to paramagnetic relaxation enhancement (Figure 4c). As observed in our glycine experiments, AsymPol-POK is much more efficient in reducing the *T*_1_(^1^H) times compared to AMUPol. At a concentration of 20 mM AsymPol-POK, *T*_1_(^1^H) is reduced to 0.6 s. This enables recycle delays close to the hardware limit, and further reduction of *T*_1_(^1^H), e.g., by using even higher concentrations, is therefore not needed. In contrast, at 20 mM AMUPol, the *T*_1_(^1^H) is still 2.4 s.

The sensitivity that takes the effect of *T*_1_(^1^H), MW on/off-enhancement and depolarization into account is shown in Figure 4d) and improves with increasing concentration of the polarizing agents. For all concentrations the samples with AsymPol-POK had higher sensitivity than the samples with AMUPol, similar to what we observed in the glycine series. A higher degree of deuteration of the DNP matrix would probably improve the sensitivity of the samples containing AMUPol. We did not continue sample optimization in this direction due to two reasons. First, in our glycerol series the samples with AsymPol-POK showed a slightly better sensitivity than the samples with AMUPol in the highly deuterated matrix. Second, below we show that AsymPol-POK has a much weaker effect on the coherence lifetimes and is therefore preferred.

To experimentally visualize the effect of the sensitivity, we recorded ^13^C-CP spectra of each sample for 10 min with a recycle delay of 1.3 *T*_1_(^1^H). Due to the differences in *T*_1_(^1^H), a different number of scans was recorded during this time. The spectra were then scaled to have the same noise by the factor: (number of scans)^0.5^. The signal intensity thus obtained was normalized to the sensitivity obtained for the sample without any radical (Figure 4g), demonstrating the large dependency of the sensitivity on the type and concentration of the polarizing agent. These intensities compare well with the calculated sensitivities in Table 1.

Based on the aforementioned data and assuming that the degree of deuteration of the matrix is low, one might conclude that a good strategy to obtain the best DNP performance on protein-detergent micelles would be to use high concentrations of the polarizing agent, ideally Asympol-POK, even above 20 mM. However, one factor that contributes to the increase in sensitivity is the reduction in the *T*_1_(^1^H) time, which results in a reduced recycle delay. At the highest AsymPol-POK concentration of 20 mM used here, the optimal recycle delay of 1.3 *T*_1_(^1^H) corresponds to 0.8 s. This is already very close or, depending on the experiment, even beyond the duty cycle of the DNP MAS probe, as experiments are usually performed using high-power proton decoupling. A further reduction in ^1^H-*T*_1_ time would therefore not be translated into a higher number of scans per time.

In addition to sensitivity, it is important to consider the linewidths and the coherence lifetimes of the samples. In the fully labeled samples used here, the linewidth observed in the one-dimensional spectra is a sum of many lines and does not reflect the linewidth of a certain site. Therefore, we measured the homogeneous *T*_2_-time using the Hahn echo experiment. The *T*_2_-times are closely related to the coherence lifetimes and *T*_1ρ_-times, which are important when performing multidimensional experiments. The results are visualized in Figure 4e,f for the ^13^C carbonyl and ^15^N amide signals, respectively. Both *T*_2_-times correlate strongly with the concentration of the polarizing agent and are reduced significantly at all concentrations. The effect is stronger for AMUPol, but AsymPol-POK also reduces the Hahn echo *T*_2_-times. Thus, the right choice of radical concentration takes into account the importance of linewidth and coherence lifetimes in the envisioned application.

Based on these data, we then decided to use 10 mM AsymPol-POK for our study of CB_2_, which represents a compromise between highest sensitivity and a small effect on *T*_2_. For experiments that require a maximum coherence lifetime, a lower AsymPol-POK concentration is advisable. Figure 4h shows the ^13^C cross-polarization spectrum of MV^TM7^-CB_2_ + CP-55,940 in detergent micelles in the presence of 10 mM AsymPol-POK, with and without microwave irradiation. An enhancement of 66 was observed, similar to what we obtained on the KR2 samples. The low degree of labeling and the difficulty in obtaining a sufficient amount of GPCR prevented the determination of the quenching factor and, therefore, the sensitivity, which requires the measurement of spectra without a polarizing agent. Table S2 shows the enhancements and ^1^H-*T*_1_ times of all CB_2_ samples used in this study.

### DNP Unique Pair Experiments on CB_2_ with the Agonist CP-55,940

**Figure 5:**
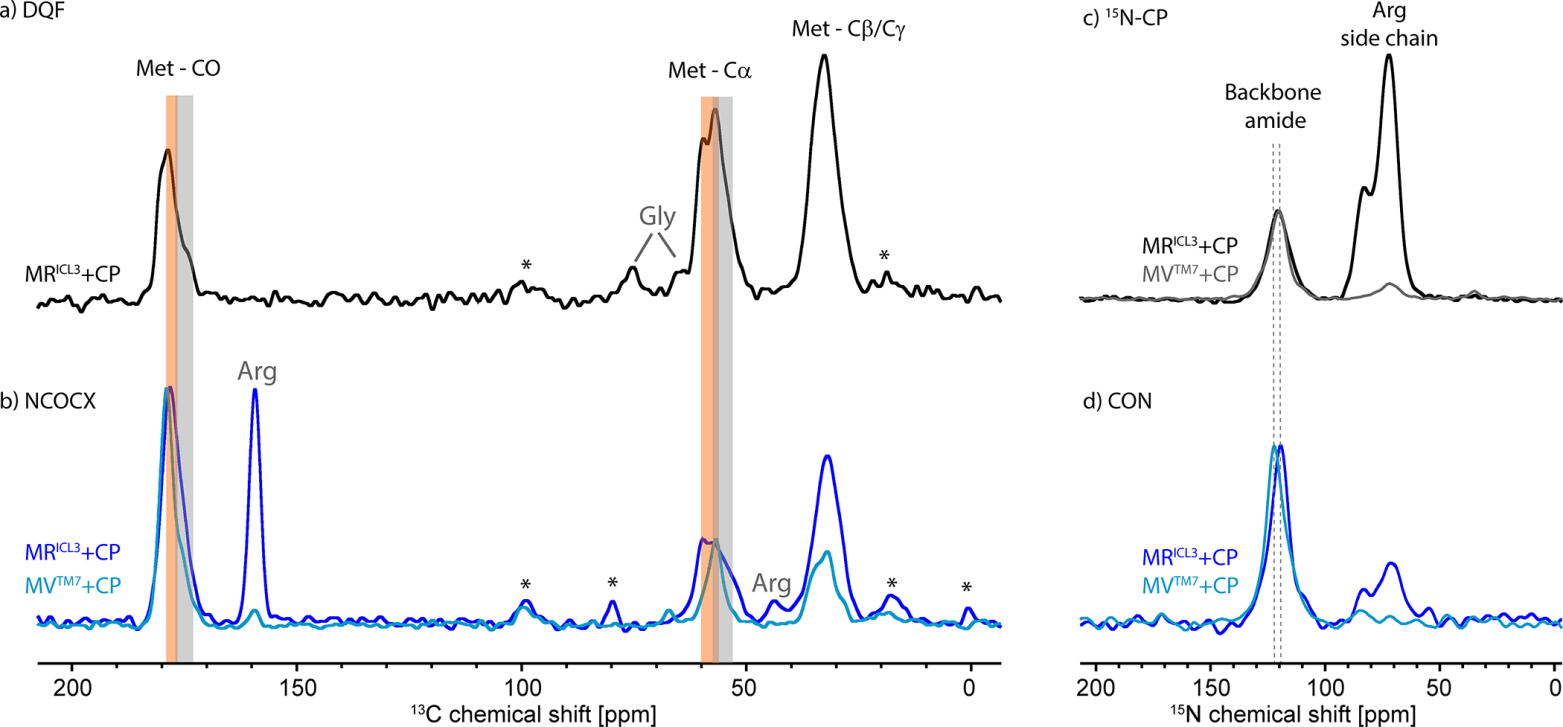
(a) Double quantum filtered spectrum of MR^ICL3^-CB_2_ + CP (ns = 128), showing signals from all methionine residues. Spinning sidebands are labeled with * and natural abundance glycerol signals with Gly. (b) NCOCX spectra showing the signals of a single methionine residue in the MR^ICL3^-CB_2_ + CP (ns = 186,496) and MV^TM7^-CB_2_ + CP (ns = 147,456) pair in dark blue and light blue, respectively. Orange bars indicate the region most likely found for methionine in alpha-helical regions of a protein, and gray bars indicate chemical shift regions in non-alpha-helical regions. Spinning sidebands are labeled with * and signals due to the ^15^N-labeling of the arginine side chain with Arg. (c) ^15^N-CP spectra of MR^ICL3^-CB_2_ + CP (ns = 128) and MV^TM7^-CB_2_ + CP (ns = 128), in black and gray, respectively. (d) CON spectra showing the signals of the single amide in the MR^ICL3^-CB_2_ + CP (ns = 65,536) and MV^TM7^-CB_2_ + CP (ns = 40,960) pair in dark blue and light blue, respectively. The dashed lines indicate the peak maxima of the unique pair amides.

First, we recorded a ^13^C double-quantum filtered (DQF) ^13^C spectrum on MR^ICL3^-CB_2_ in the presence of the full agonist CP-55,940 (MR^ICL3^-CB_2_ + CP) that greatly enhances the stability of the receptor.^42^ The spectrum showed exclusively signals from the 10 methionine residues (Figure 5a). Especially on the Cα signals, a non-uniform chemical shift distribution was seen. From the DQ filtered spectrum alone, it cannot be concluded if this results from the different chemical shifts of the 10 different residues or if the single residues are subject to conformational heterogeneity. Similarly, broadening was also observed for the carbonyl line shape. The Cβ and Cγ signals overlapped and were not further analyzed here. Due to the double quantum filtering, no signals were observed from the Cε sites.

In contrast, the unique pair-filtered NCOCX spectra (Figure 5b) showed signals from just a single methionine residue, in MR^ICL3^-CB_2_ + CP (dark blue) and MV^TM7^-CB_2_ + CP (light blue), respectively. The spectrum recorded on MR^ICL3^-CB_2_ + CP showed additional signals at around 160 and at 43 ppm that were caused by the one-bond correlation of the fully ^15^N isotope labeled arginine side chain with the Cζ and Cδ arginine carbon atoms at natural abundance. All the arginine residues in the protein contribute to these signals and, therefore, do not yield site resolved information.

For MR^ICL3^-CB_2_ + CP broad lines were observed comparable to the DQF spectrum, showing that M237 in MR^ICL3^-CB_2_ + CP samples a large conformational space. The line of the Cα signal showed a heterogeneous shape, indicating the presence of different populations. The situation was different for the signals observed in the NCOCX spectrum of the MV^TM7^-CB_2_ + CP sample. These signals were narrower compared to the DQF spectrum and showed that the conformational space of M293 in MV^TM7^-CB_2_ + CP is significantly restricted, compared to M237 in MR^ICL3^-CB_2_ + CP.

CO, Cα, and Cβ chemical shifts depend strongly on the dihedral angles and thus the secondary structure of the protein backbone. Fritzsching et al.^18^ have correlated the chemical shift information from the BMRB with the structural information provided by the PDB using the PACSY database^56^ and designed the python tool PLUQ to make this information usable. The tool predicts probabilities of a certain chemical shift to originate from a specific secondary structure. For methionine, we analyzed the Cα and CO chemical shifts with PLUQ and then defined two chemical shift ranges: A propensity for alpha helical secondary structure above 20% (Cα: 56–60 ppm, CO: 179–176.5 ppm) and a propensity of a non-alpha helical structure (random coil or beta sheet) above 20% (Cα: 57.5–53 ppm, CO: 177–173 ppm). These regions are visualized in Figure 5a,b as orange and gray bars, respectively.

As expected for a GPCR, our observed signals overlapped well with the alpha-helical chemical shift region. However, with the exception of Cα(M293), the signals were broader than the total chemical shift range that is analyzed by the PLUQ tool, including non-alpha-helical shifts. The chemical shifts in the PACSY database are derived from spectra recorded at room temperature. Under this condition, the chemical shifts are averaged due to fast dynamics. In the frozen state, fast as well as slow motions are quenched and the full chemical shift range becomes observable. We conclude that M293 in TM7 is, most of the time, in the alpha-helical fold, whereas M237 in ICL3 samples a large conformational space that includes alpha-helical as well as non-alpha-helical conformations.

For the selectively labeled samples, we also recorded spectra with a magnetization transfer from the carbonyl to the amide backbone, showing an amide signal for the first amino acid of the respective unique pair. The CON spectra for the two samples, MR^ICL3^-CB_2_ + CP and MV^TM3^-CB_2_ + CP, are shown in Figure 5d along with the ^15^N-CP spectra (Figure 5c) that show the ^15^N signals of all arginine or all valine residues, respectively. The spectra differed in their chemical shift but had a similar linewidth, which is narrower than the distribution of all arginine or valine amide signals. The larger conformational space in ICL3, compared to TM7, was not observed in the amide chemical shifts as these are less sensitive to the backbone dihedral angles.

### Effect of Different Ligands

**Figure 6:**
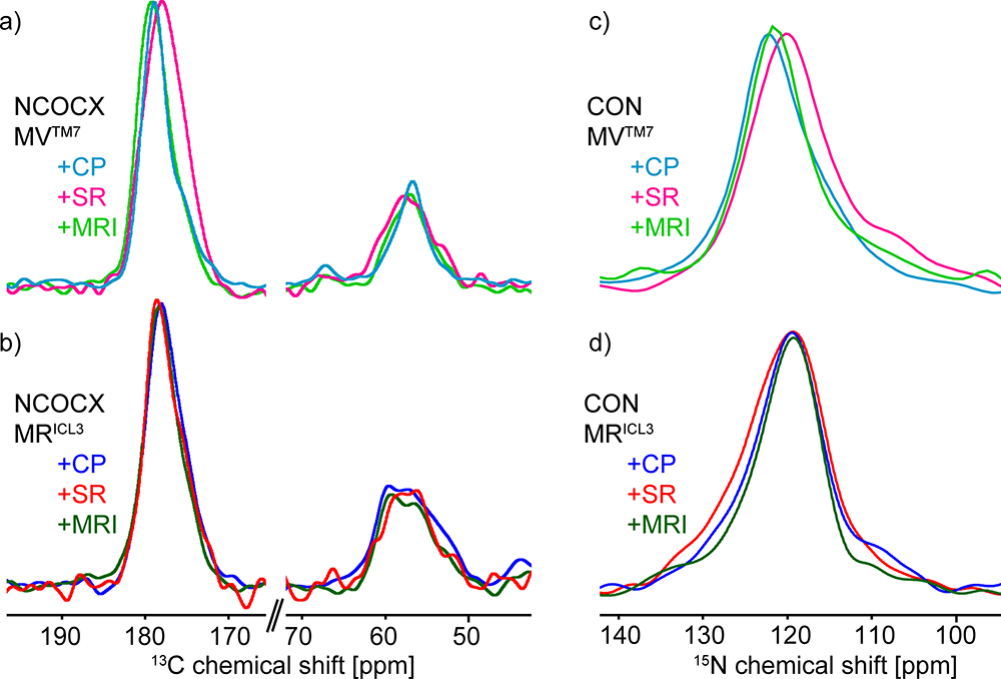
(a) NCOCX spectra of MV^TM7^-CB_2_ with different ligands (CP: ns = 147,456, SR: ns = 153,216, MRI: ns = 163,640). (b) NCOCX spectra of MR^ICL3^-CB_2_ with different ligands (CP: ns = 186,496, SR: ns = 40,960, MRI: ns = 169,088). (c) CON spectra of MV^TM7^-CB_2_ with different ligands (CP: ns = 40,960, SR: ns = 98,304, MRI: ns = 81,920). (d) CON spectra of MR^ICL3^-CB_2_ with different ligands (CP: ns = 65,536, SR: ns = 40,960, MRI: ns = 62,464).

The conformational ensemble of a GPCR might vary depending on the state of the protein. To probe this, we prepared samples with the two selectively labeled unique pairs in the partial agonist and antagonist bound state by replacing the agonist CP-55,940 with MRI-2653 and SR-144,528, respectively (Figure 1a) and thus analyzed 4 more samples: MR^ICL3^-CB_2_ + MRI, MR^TM7^-CB_2_ + MRI, MV^ICL3^-CB_2_ + SR, and MV^TM7^-CB_2_ + SR. The NCOCX and CON spectra off these samples are shown in Figure 6.

The line shape of CO, Cα, and N in MR^ICL3^-CB_2_ showed little effect on the type of ligand and remained broad, indicating a large conformational space in all three preparations. In contrast, the MV^TM7^-CB_2_ line shape was sensitive to the type of ligand. This effect was most pronounced on the CO line, which showed significant broadening upon binding to the antagonist SR-144,528, whereas replacing CP-55,940 by MRI-2653 had no such effect. Cα and N signals of MV^TM7^-CB_2_ + SR were also broadened compared to MV^TM7^-CB_2_ + CP and MV^TM7^-CB_2_ + MRI. The CO and N signals of MV^TM7^-CB_2_ + SR not only showed broadening but also a slight change in chemical shift. The direction of the shift agreed with a higher propensity of this site to be in a non-alpha helical conformation.

Next, we wanted to exclude the possibility that the observed line broadening was caused by paramagnetism of the polarizing agent. Such broadening is homogeneous in nature, whereas line broadening due to freezing of a large conformational space is inhomogeneous and can be refocused. To analyze which line broadening effect is dominant in our samples, we recorded NCO-Hahn echo experiments on MV^TM7^-CB_2_ + CP, MV^TM7^-CB_2_ + SR, and MV^TM7^-CB_2_ + MRI (Figure S3). The carbonyl Hahn echo *T*_2_-times obtained on all three samples were more than an order of magnitude longer than what would be expected from the observed linewidth, which thus is inhomogeneous in nature and paramagnetic effects do not significantly affect the observed line shapes.

Based on these data, we conclude that formation of the inactive conformation of CB_2_ by antagonist binding results in a change in TM7 toward a larger conformational freedom, compared to the active state. Activation of CB_2_ has been shown to depend on the membrane environment and especially on the presence of cholesterol in a way that some ligands can act as an agonist in one environment and as inverse agonist on another one.^29^ MRI-2653 is such a ligand and in the absence of cholesterol has been observed to act as a partial agonist. This is in good agreement with our observation. The NMR spectra of our cholesterol-free CB_2_-MRI-2653 samples were very similar to the spectra observed on CB_2_ bound to the full agonist CP-55,940, thus indicating that MRI-2653 functioned as an agonist in our preparations.

### Chemical Shift Distributions

**Figure 7:**
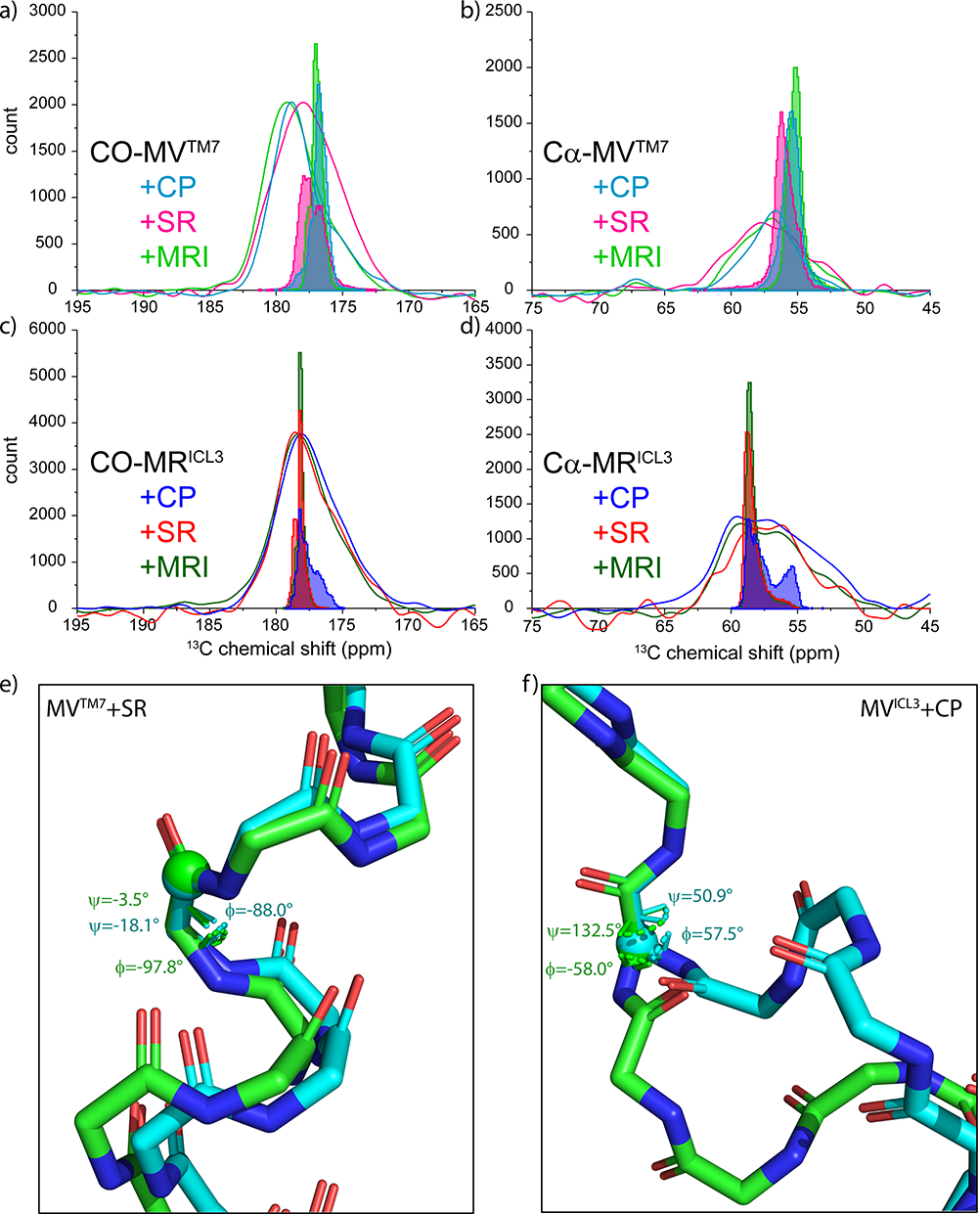
Comparison of CO (a, c) and Cα (b, d) regions of the NCOCX spectra of ligand-bound MV^TM7^-CB_2_ and MR^ICL3^-CB_2_ with predicted chemical shift distributions obtained from the MD traces. The spectra are arbitrarily scaled and the *y* axis shows the number of counts from the MD traces. (e) Region around CO (sphere) in M293 in TM7 in the presence of SR-44,528 with the dihedral angles of M293 in two snapshots of the MD trajectories: green and cyan correspond to a CO chemical shift prediction of 176.6 and 177.6 ppm, respectively. (f) Region around Cα (sphere) in M237 in ICL3 in the presence of CP-55,940 with the dihedral angles of M237 in two snapshots of the MD trajectories: green and cyan correspond to a Cα chemical shift prediction of 55.4 ppm and 58.4 ppm, respectively.

Finally, we wanted to compare our experimental chemical shift distribution with the distribution that is expected based on the available cryo-EM structure. Therefore, we built a model using the cryo-EM structure 6PT0,^9^ replaced the ligand by CP-55,940, SR-44,528, and MRI-2653, respectively, and subjected each model to an unbiased all-atom equilibrium MD simulation of 500 ns, obtaining 25,000 frames in each case. For each frame, the methionine chemical shifts of the two unique pairs was predicted using SHIFTX.^57^ The prediction is based on a semiempirical approach taking ring current, electric field, hydrogen bond, and solvent effects into account. The obtained chemical shift distribution in each of the 6 simulations are narrower than the experimental line shapes, Figure 7a–d. This shows that motions on timescales longer than the 500 ns of the MD trajectory are frozen in our system. In TM7, the calculated chemical shift distributions, Figure 7a,b, for the CP-55,940 and MRI-2653 ligands are similar. In contrast, the simulation with SR-44,528 shows a bimodal chemical shift distribution. A similar behavior is seen in the experimental data with the difference that the higher populated chemical shift range is at higher frequencies in contrast to the simulations. Interestingly, in ICL3 the samples with the ligands SR-44,528 and MRI-2653 show similarly narrow distributions, whereas the sample with CP-55,940 covers an additional chemical shift range. Experimentally, this effect is less pronounced.

Chemical shifts generally depend on the environment of a nucleus and not directly on temperature or aggregation state. However, chemical shift prediction tools such as SHIFTX are usually benchmarked against solution NMR data, which are averaged on the NMR timescale (up to ms). Therefore, conformations with extreme chemical shifts with a short lifetime or low probability are usually not directly measured. This results in a lack of these chemical shifts in semiempirical approaches. In contrast, our data on frozen samples contain such chemical shifts as most dynamics is frozen, resulting in a broader linewidth than predicted from SHIFTX for all our signals. The situation is even more extreme when using machine learning based algorithms such as SHIFTX2.^58^ They are even more tailored to generate the time averaged chemical shifts found in the solution NMR training sets and result in extremely narrow lines for our MD-trajectories, see Figure S4. To improve such chemical shift predictions and to correlate the structures in the MD-trajectories with the experimental shifts, chemical shift prediction has to be improved, ideally using *ab initio* approaches. However, currently, these are time-consuming and difficult to run on whole MD-trajectories. Simulations that reproduce the experimental line shapes can be used to understand the conformational space present in a sample. To illustrate this, we used the MD simulations, which indicate the presence of two discrete groups of conformations by showing bimodal distribution: CO-MV^TM7^ + SR-44,538 and Cα-MR^ICL3^ + CP-55,940. We selected two structures of the trajectories representative of the maximum of each of the two maxima in the chemical shift distributions and compared them, Figure 7e,f. TM7 in the presence of SR-44,538 shows slight variations in the protein backbone but no disruption in the helical structure, whereas ICL3 in the presence of CP-55,940 shows large differences in the conformation between the two structures. In theory, it should be possible to find a structural ensemble that reproduces our observed line shapes and thus gives quantitative insight into the dynamics of a GPCR. However, further improvements in chemical shift predictions as well as in covering the ms time range in simulations are needed.

## Summary and Conclusions

The aim of this study was to optimize and apply experimental conditions for DNP-enhanced MAS NMR for probing ligand binding effects in a detergent solubilized GPCR. We have systematically analyzed DNP sample preparations for protein detergent micelles. The degree of deuteration as well as the glycerol content of the glassy matrix has been optimized on a model system. Comparison of AMUPol and AsymPol-POK as polarizing agents revealed several advantages of AsymPol-POK when studying protein detergent micelle preparations, especially when signal-to-noise ratio and coherence lifetimes are important. Additionally, we showed that AsymPol-POK can tolerate a much higher level of protonation in the DNP matrix. Another interesting observation is that AMUPol enhancements are still reasonable when the glycerol content is drastically reduced. Reducing the glycerol content can be important when the sample of interest does not tolerate high amounts of glycerol or when the concentration of protein needs to be increased to improve sensitivity.

After optimizing the sample preparation, we studied the CB_2_ receptor. We strategically placed unique pair labels at two different positions in the protein. We observed differences in the line width of the signals depending on the site in the protein and on the type of ligand, resulting from differences of the conformational space available. The site in TM7 showed a differential response to these ligands whereas ICL3 samples a large conformational space in the presence of all tested ligands. The advantage of the method is that the introduced isotope labels do not change the properties of the protein as the sequence and all side chains are preserved. In addition, the low working temperatures enable the study of proteins with limited stability. The full conformational space is observed as all sites are in the slow-motion regime and no signal is lost to states invisible due to intermediate motion. In contrast to the ^19^F labels applied in other NMR approaches,^13^ our labels probe the backbone conformation of the protein directly and should not be influenced by changes in solvation that strongly influence commonly used ^19^F labels, which are usually positioned away from the backbone.

Our work shows that conformations with extreme chemical shift values are populated in our samples emphasizing the large dynamics present in GPCR. In contrast, in a recent work on frozen viral capsids,^19^ the experimental chemical shift distributions were much narrower and resembled the distributions obtained by SHIFTX. Therefore, it will by highly interesting to analyze MD trajectories with improved chemical shift prediction algorithms to yield information on the short lived or sparsely populated conformations in GPCRs, which are hidden to most structural techniques but very likely functionally relevant.

The described principle of probing the conformational space qualitatively, by freezing the whole ensemble can be easily transferred to more complex systems, e.g., complexes of GPCRs with G-proteins or arrestins. There is no intrinsic size limit to the method, and adoption to other membrane mimetic environments, e.g., lipid bilayers, is also possible. It will be interesting to investigate if the conformational space in ICL3 of CB_2_ changes in the presence of the G-protein.

## Supporting Information Available

Table S1: DNP and relaxation parameters of all 1-^13^C-glycine samples; Table S2: enhancement and *T*_1_(^1^H) data of all CB_2_ samples; Figure S1: nucleotide sequence of the CB_2_ expression construct; Figure S2: bound MRI in the NMR sample; Figure S3: with *T*_2_ measurements on CB_2_; Figure S4: comparison of the chemical shift distributions obtained from the MD models using SHIFTX and SHIFTX2, respectively (PDF)
