# Supplementary material for: Probing the Conformational Space of the Cannabinoid Receptor 2 and a Systematic Investigation of DNP-Enhanced MAS NMR Spectroscopy of Proteins in Detergent Micelles
Source: ACS Omega. 2023 Aug 28;8(36):32963–76. doi: 10.1021/acsomega.3c04681 (PMC10500644; doi:10.1021/acsomega.3c04681)
Supplement: Supplementary file 1 — ao3c04681_si_001.pdf [file ao3c04681_si_001.pdf]

Supplementary Information

Probing the conformational space of the cannabinoid receptor 2 and a systematic investigation of DNP-enhanced MAS NMR spectroscopy of proteins in detergent micelles

Johanna Becker-Baldus[1*], Alexei Yeliseev[2*], Thomas T. Joseph[3], Snorri Th. Sigurdsson[4], Lioudmila Zoubak[2], Kirk Hines[2], Malliga R. Iyer[5], Arjen van den Berg[6], Sam Stepnowski[6], Jon Zmuda[6], Klaus Gawrisch[2], Clemens Glaubitz[1*]

[1]Institute of Biophysical Chemistry and Centre of Biomolecular Magnetic Resonance, Goethe University Frankfurt, Max-von-Laue-Str. 9, 60438 Frankfurt, Germany.

[2]National Institute on Alcohol Abuse and Alcoholism, National Institutes of Health, Bethesda, MD 20852, USA.

[3]Department of Anesthesiology and Critical Care, Perelman School of Medicine, University of Pennsylvania, Philadelphia, PA 19104, USA.

[4]University of Iceland, Department of Chemistry, Science Institute, Dunhaga 3, 107 Reykjavik, Iceland.

[5]Section on Medicinal Chemistry, National Institute on Alcohol Abuse and Alcoholism, National Institutes of Health, Bethesda, MD 20852, USA

[6]ThermoFisher Scientific, 7335 Executive Way, Frederick, MD 21704, USA

Table S1: Microwave on (MW on) vs. Microwave off (MW off) enhancement and $^1H(T_1)$ times on 1-^{13}C-Glycerol in different DNP matrices. All samples except the one without radical contained either 5 mM AMUPol or 5mM AsymPol-POK. Errors for T_1 correspond to double standard deviation. Samples prepared with protonated instead of deuterated glycerol are labelled with "H".

**Sample H_2O:D_2O:$^2H_8$-Glycerol (v/v/v)**	**Enhancement (MW on vs. MW off)**	**depolarization**	**$^1H(T_1)$ MW on (s)**	**$^1H(T_1)$ MW off (s)**	**Sensitivity (s^{-1})**
1:4:5 no pol. agent	1	1.00	-	56.7 ± 0.5	0.1
1:9:0 AMUPol	1	0.71	0.58 ± 0.03	0.57 ± 0.02	0.9
1:8:1 AMUPol	123	0.38	2.1 ± 0.1	1.9 ± 0.04	32.0
1:7:2 AMUPol	146	0.43	4.8 ± 0.1	4.6 ± 0.1	28.5
1:6:3 AMUPol	131	0.54	6.4 ± 0.3	6.8 ± 0.4	28.1
1:5:4 AMUPol	145	0.64	14.5 ± 0.2	14.2 ± 0.1	24.3
1:4:5 AMUPol	177	0.54	13.0 ± 0.2	13.2 ± 0.3	26.3
1:3:6 AMUPol	177	0.59	16.6 ± 0.3	16.3 ± 0.2	25.8
1:2:7 AMUPol	204	0.56	15.8 ± 0.3	16.3 ± 0.2	28.8
1:1:8 AMUPol	207	0.55	16.7 ± 0.3	17.1 ± 0.3	28.0
1:0:9 AMUPol	217	0.49	15.4 ± 0.2	15.5 ± 0.6	27.1
5:0:5 AMUPol	84	0.81	12.8 ± 0.1	12.9 ± 0.1	18.9
1:4:5H AMUPol	82	0.86	12.9 ± 0.2	13.2 ± 0.2	19.7
5:0:5H AMUPol	53	0.91	11.8 ± 0.1	11.8 ± 0.1	14.2
1:4:5 AsymPol-POK	68	0.81	2.84 ± 0.05	2.99 ± 0.03	32.8
5:0:5 AsymPol-POK	64	0.89	2.60 ± 0.02	3.36 ± 0.04	35.7
5:0:5H AsymPol-POK	49	0.59	4.44 ± 0.09	5.5 ± 0.1	13.7

Table S2: Enhancements and $^{1}H(T_1)$-times of the CB_2 samples used in this work. $^{1}H(T_1)$ of MR^{ICL3}-SR is significantly longer than the $^{1}H(T_1)$-times of the other 5 samples and at the same time shows a lower enhancement. We assume that the sample contained less than 10 mM AsymPol-POK due to pipetting errors. We did not attempt to add more radical to avoid unpacking of the valuable sample.

Sample	Enhancement (MW on vs. MW off)	$^{1}H(T_1)$ MW on [s]
MV^{TM7}-CP	66	1.6 ± 0.2
MV^{TM7}-SR	64	1.3 ± 0.2
MV^{TM7}-MRI	72	1.7 ± 0.2
MR^{ICL3}-CP	64	1.0 ± 0.1
MR^{ICL3}-SR	52	2.7 ± 0.2
MR^{ICL3}-MRI	64	1.2 ± 0.1

bp 163-1245 of NM_001841.3, N-terminal twin-Streptag, C-terminal 10-Histag

GCCACCATGGGATCGTGGTCGCATCCGCAGTTTGAAAAAGGATCGGGCGGCGCT
AGCTGGTCGCATCCGCAGTTTGAAAAAGGCGGCGGATCCGAGGAATGCTGGGTG
ACAGAGATAGCCAATGGCTCCAAGGATGGCTTGGATTCCAACCCTATGAAGGAT
TACATGATCCTGAGTGGTCCCCAGAAGACAGCTGTTGCTGTGTTGTGCACTCTTC
TGGGCCTGCTAAGTGCCCTGGAGAACGTGGCTGTGCTCTATCTGATCCTGTCCTC
CCACCAACTCCGCCGGAAGCCCTCATACCTGTTCATTGGCAGCTTGGCTGGGGCT
GACTTCCTGGCCAGTGTGGTCTTTGCATGCAGCTTTGTGAATTTCCATGTTTTCCA
TGGTGTGGATTCCAAGGCTGTCTTCCTGCTGAAGATTGGCAGCGTGACTATGACC
TTCACAGCCTCTGTGGGTAGCCTCCTGCTGACCGCCATTGACCGATACCTCTGCC
TGCGCTATCCACCTTCCTACAAAGCTCTGCTCACCCGTGGAAGGGCACTGGTGAC
CCTGGGCATCATGTGGGTCCTCTCAGCACTAGTCTCCTACCTGCCCCTCATGGGA
TGGACTTGCTGTCCCAGGCCCTGCTCTGAGCTTTTCCCACTGATCCCCAATGACTA
CCTGCTGAGCTGGCTCCTGTTCATCGCCTTCCTCTTTTCCGGAATCATCTACACCT
ATGGGCATGTTCTCTGGAAGGCCCATCAGCATGTGGCCAGCTTGTCTGGCCACCA
GGACAGGCAGGTGCCAGGAATGGCCCGAATGAGGCTGGATGTGAGGTTGGCCAA
GACCCTAGGGCTAGTGTTGGCTGTGCTCCTCATCTGTTGGTTCCCAGTGCTGGCC
CTCATGGCCCACAGCCTGGCCACTACGCTCAGTGACCAGGTCAAGAAGGCCTTTG
CTTTCTGCTCCATGCTGTGCCTCATCAACTCCATGGTCAACCCTGTCATCTATGCT
CTACGGAGTGGAGAGATCCGCTCCTCTGCCCATCACTGCCTGGCTCACTGGAAGA
AGTGTGTGAGGGGCCTTGGGTCAGAGGCAAAAGAAGAAGCCCCGAGATCCTCAG
TCACCGAGACAGAGGCTGATGGGAAAATCACTCCGTGGCCAGATTCCAGAGATC
TAGACCTCTCTGATTGCCACCATCACCATCACCATCACCATCACCATTGA

KOZAK

TST

CB2

10HIS

Figure S1: Nucleotide sequence of CB_2 expression construct.

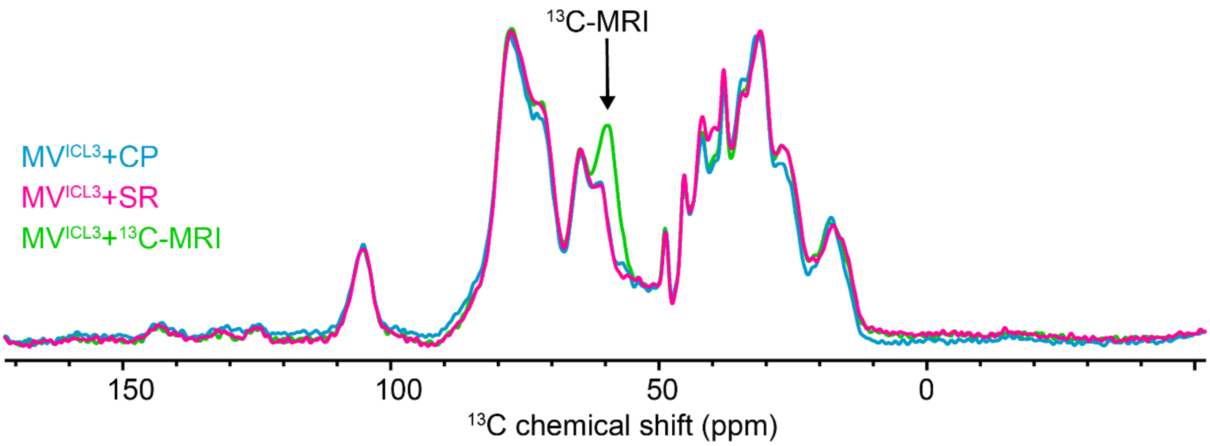

Figure S2: ^{13}C CP MAS spectra of the three different MV^{ICL3} samples. The signal from the ^{13}C-MRI ligand is indicated and shows the presence of the ligand in the sample.

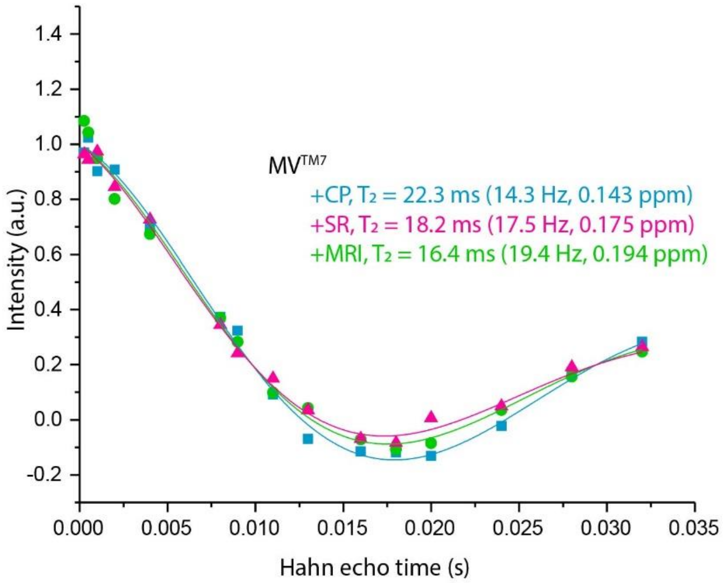

Figure S3: Hahn echo T_2 times of the NCO signal in the MV^{TM7} labelling schemes for the three different ligands. T_2 was obtained by fitting the data to: $f(t) = A \exp(-t/T_2) \cos(J \pi t) + y0$ with $J(CO\text{-}C\alpha) = 51$ Hz. The corresponding homogenous line width is given in the figure in brackets in Hz and ppm and was calculated using: line width = $1/(\pi T_2)$.

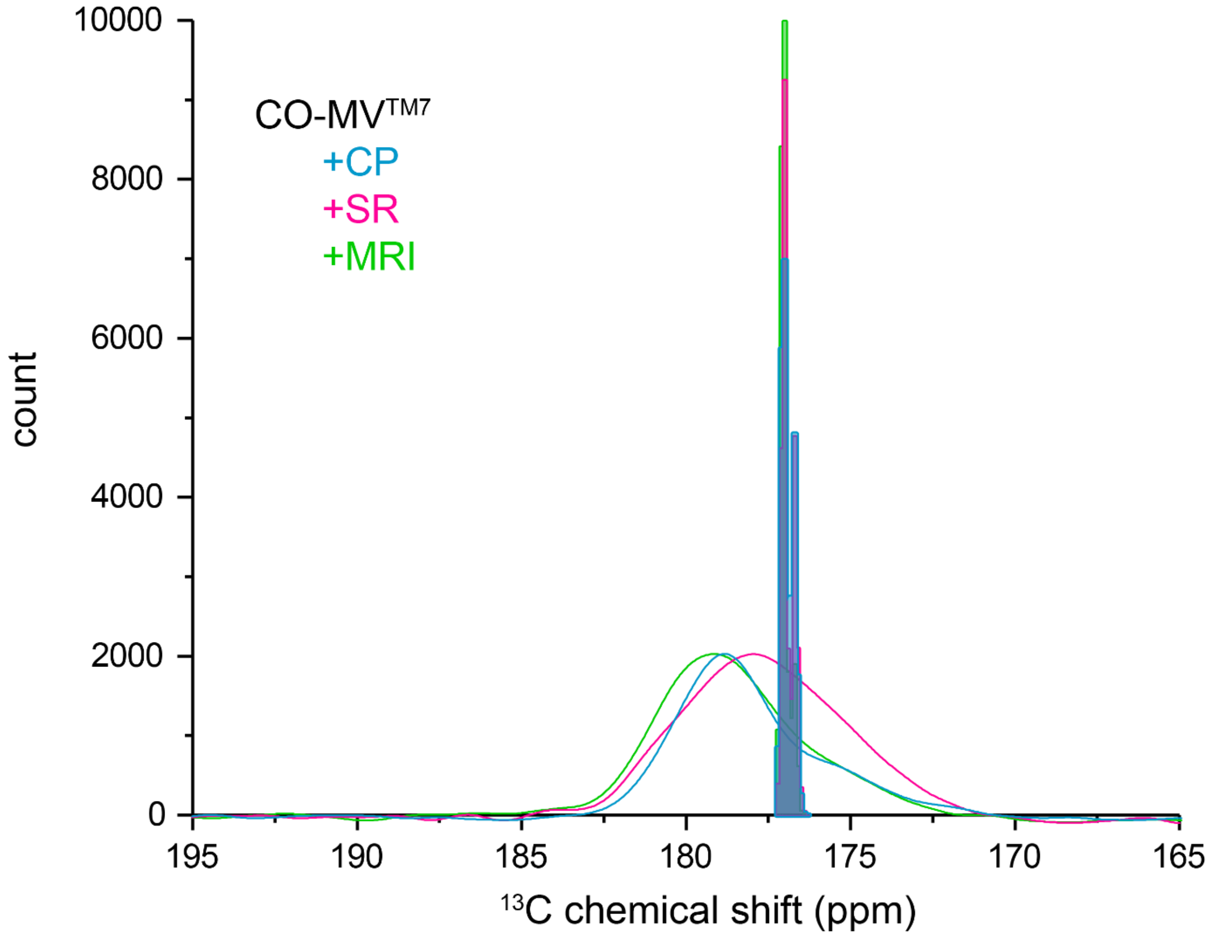

Figure S4: Chemical shift distributions obtained with SHIFTX2 from MD traces for CO-MV^{TM7} together with the experimental line shapes (lines).
